# Green Synthesis of Hexagonal Silver Nanoparticles Using a Novel Microalgae *Coelastrella aeroterrestrica* Strain BA_Chlo4 and Resulting Anticancer, Antibacterial, and Antioxidant Activities

**DOI:** 10.3390/pharmaceutics14102002

**Published:** 2022-09-21

**Authors:** Reham Samir Hamida, Mohamed Abdelaal Ali, Zakiah Nasser Almohawes, Hadil Alahdal, Maha Abdullah Momenah, Mashael Mohammed Bin-Meferij

**Affiliations:** 1 Molecular Biology Unit, Department of Zoology, Faculty of Science, Alexandria University, Alexandria 21321, Egypt; 2 Biotechnology Unit, Department of Plant Production, College of Food and Agriculture Science, King Saud University, Riyadh 11451, Saudi Arabia; 3 Department of Biology, College of Science, Princess Nourah bint Abdulrahman University, Riyadh 11671, Saudi Arabia; 4 Histopathology Unit, Research Department, Health Sciences Research Center (HSRC), Princess Nourah bint Abdulrahman University, Riyadh 11671, Saudi Arabia

**Keywords:** biofabrication, hexagonal, breast, liver, colon cancer, DPPH, pathogenic bacteria

## Abstract

Microalgae-mediated synthesis of nanoparticles (NPs) is an emerging nanobiotechnology that utilizes the biomolecular corona of microalgae as reducing and capping agents for NP fabrication. This study screened a novel microalgal strain for its potential to synthesize silver (Ag)-NPs and then assayed the biological activities of the NPs. *Coelastrella aeroterrestrica* strain BA_Chlo4 was isolated, purified, and morphologically and molecularly identified. Chemical composition of the algal extract was determined by GC-MS analysis. Ag-NPs were biosynthesized by *C. aeroterrestrica* BA_Chlo4 (C@Ag-NPs) and characterized using various techniques. Antiproliferative activity and the biocidal effect of C@Ag-NPs, *C. aeroterrestrica* algal extract, and chemically synthesized Ag-NPs (Ch@Ag-NPs) were explored, and the scavenging activity of C@Ag-NPs against free radicals was investigated. C@Ag-NPs were hexagonal, with a nanosize diameter of 14.5 ± 0.5 nm and a maximum wavelength at 404.5 nm. FTIR and GC-MS analysis demonstrated that proteins and polysaccharide acted as capping and reducing agents for C@Ag-NPs. X-ray diffraction, energy diffraction X-ray, and mapping confirmed the crystallinity and natural structure of C@Ag-NPs. The hydrodynamic diameter and charge of C@Ag-NPs was 28.5 nm and −33 mV, respectively. C@Ag-NPs showed significant anticancer activity towards malignant cells, with low toxicity against non-cancerous cells. In addition, C@Ag-NPs exhibited greater antioxidant activity and inhibitory effects against Gram-positive and -negative bacteria compared with the other tested treatments. These findings demonstrate, for first time, the potential of a novel strain of *C. aeroterrestrica* to synthesize Ag-NPs and the potent antioxidant, anticancer, and biocidal activities of these NPs.

## 1. Introduction

Nanotechnology is currently transforming the therapeutic and diagnostic fields of many diseases. In addition, the influence of this technology is extending beyond the medical sector to many other fields including agriculture, electronics, industry, and pharmaceuticals. Nanotechnologies involve the synthesis of nanostructures with a diameter of about one-thousandth of the thickness of a hair. These nanostructures substantially impact global morbidity and mortality [1,2]. Many nanosystems have been approved by the Food and Drug Administration (FDA) as chemotherapeutics, bioimaging tools, and nutritional supplements [3,4]. There are three main methods for fabricating nanoparticles (NPs), including physical, chemical, and biological approaches [5,6].

Green nanobiotechnology is a promising approach of nanotechnology to biofabricate different nanostructures using natural sources, including cells of plants, bacteria, algae, microalgae, cyanobacteria, and fungi, and their biomolecules such as vitamins, enzymes, pigments, etc. [7,8,9,10]. The theory behind using natural sources is that these cells contain reducing and stabilizing agents (e.g., enzymes, polysaccharides, proteins, etc.) as natural alternatives for chemical and physical methods responsible for synthesizing NPs [11].

Recently, biofabrication has become an attractive approach because it is an ecofriendly method with low or no hazardous yield, and the biogenic NPs produced by this method show potent physicochemical and biological features such as having smaller size with large surface area, high stability, thermal and electrical conductivity, low toxicity, and a high tendency to be loaded by other materials including antibiotics and anticancer drugs [12,13]. These properties enable these NPs to be applied as biosensors, therapeutic agents, drug delivery vehicles, catalysts, etc. [14,15,16,17].

Microalgae are considered a sustainable alternative source for NPs synthesis [10]. The ability of these microorganisms to survive in diverse and extreme environmental conditions facilitates their use as potent bioagents to eliminate heavy metal pollutants [18]. Microalgae use low concentrations of metals in their niches to perform many cellular functions, including photosynthetic electron transfer, as cofactors in enzymatic reactions, and N_2_ assimilation. However, a high concentration of metals could have toxic effects on the morphology and functions of microalgae. Therefore, to mitigate the heavy metal toxicity, microalgal cells secrete biomolecules such as metal chelating agents that convert these heavy metals into nanosized metal nuclei [18,19,20]. These potentialities qualify microalgae as green entities for the synthesis of NPs [21,22,23]. Members of the genus *Coelastrella* are promising microalgae in biotechnological and industrial applications due to their fatty acid and pigment contents as well as their ability to bioremediate heavy metals. *Coelastrella aeroterrestrica* was taxonomized and morphologically described for the first time by Tschaikner et al. [24]. The authors isolated the microalgae from soil in Austria and examined it using light and scanning electron microscopes (SEM). The strain had ribs on its surface that appeared only under SEM and did not have vacuoles. In 2022, *Coelastrella aeroterrestrica* strain BA_Chlo4 was deposited in the GenBank database by Russian scientist Krivina under the accession number OM471999. There are no reports on the morphological appearance and activities of *Coelastrella aeroterrestrica* BA_Chlo4, but the strain used in the current study is similar to that isolated by Krivina. This is the first report to isolate *Coelastrella aeroterrestrica* BA_Chlo4 from soil in Egypt, study the morphological appearance, and use the novel strain as a biofactory for the synthesis of Ag-NPs, including screening the application of both the algal aqueous extract and Ag-NPs as antioxidant, anticancer, and antibacterial agents.

Metallic NPs (M-NPs) with dimensions ranging from 1 to 100 nm and involving silver (Ag), gold (Au), and platinum (Pt) have significant therapeutic activities against many disorders such as cancers [25,26], diabetes [27], infectious disease [28,29], and others. For instance, Au-NPs have excellent photoacoustic and photothermal features due to their potential to absorb specific wavelengths, placing them top of the list of nano-agents used in hyperthermic cancer therapy and bioimaging [30]. Furthermore, Ag-NPs are promising therapeutic agents against pathogenic microbes and malignant cells [31,32]. There are numerous reports of Ag-NPs acting as potent biocidal agents against Gram-positive and -negative bacteria and various pathogenic fungi such as *Candida albicans* [33,34,35]. Moreover, Ag-NPs showed high therapeutic activity and selectivity against different malignant cells such as MCF-7, HepG2, Caco-2, HCT-116, and others [15,36,37]. The marked therapeutic effect of these M-NPs compared with their bulks is attributed to their physicochemical properties such as smaller size to larger surface area and surface chemistry, enabling them to easily penetrate cell membranes and interact with other organelles and vital biomolecules such as enzymes and proteins, causing intensive oxidative stress, cellular dysfunction, and finally enhancing programming cell death [10,38]. The killing mechanism of these NPs can be summarized into (i) their ability to enhance oxidative stress by increasing the formation of reactive oxygen species (ROS) inside targeted cells, which promotes the apoptosis signaling pathway; and (ii) direct interaction with cellular components and organelles causes cellular dysfunction and cell death [15,39]. The current study is the first report revealing the potentiality of the novel microalgae strain *Coelastrella aeroterrestrica* BA_Chlo4 to biofabricate Ag-NPs (C@Ag-NPs). In addition, the biological and chemical activities of both C@Ag-NPs and *C. aeroterrestrica* BA_Chlo4 algal extract against cancerous (MCF-7, MDA, HCT-116, and HepG2) and non-cancerous cells (HFS and Vero) and Gram-positive and -negative bacteria were screened and their antioxidant activities were assayed.

## 2. Materials and Methods

### 2.1. Reagents

All chemicals and kits, including chemically synthesized Ag-NPs (Ch@Ag-NPs (576832-5G), with a nanosize of <100 nm and spherical shape, 99.5% purity), 2,2-diphenyl-1-picrylhydrazyl (DPPH (D9132-1G)), and MTT (M2128-250MG) were purchased from Sigma Aldrich (St. Louis, MO, USA), and resazurin dye (20101) was from BDH chemicals (England). Cell culture materials were purchased from Gibco (Thermo Fisher Scientific, Waltham, MA, USA), while MCF-7, MDA, HCT-116, HepG2, HFS, and Vero cells were obtained from the American Type Culture Collection (ATCC, Manassas, VA, USA).

### 2.2. Methods

#### 2.2.1. Microalgae Isolation

Samples of muddy soil in Alexandria, Egypt were collected in sterile Falcon tubes (50 mL) and transported to the laboratory. The sample was then incubated in a sterilized petri dish containing BG11 media in an incubator under a fluorescence lamp (2000 ± 200 Lux) with 12:12 h dark/light cycles at ambient temperature for a week. The serial dilution method was used to purify the samples, as described by Bolch et al. [40]. Next, 50 µL of the diluted sample was inoculated on BG-11-agar plates and incubated under the same conditions. Purified colonies were grown in sterilized test tubes and examined using light microscopy to check the purity of the samples. For large-scale microalgae growth, aliquots from purified samples were grown for 15 days in 250 mL flasks containing BG11 media.

#### 2.2.2. Morphological and Molecular Identification of Microalgae

##### Light and Inverted Light Microscopy

The morphological appearance of *Coelastrella aeroterrestrica* BA_Chlo4 was identified using inverted (Thermo Fisher Scientific) and light (Novex, Holland, The Netherlands) microscopes.

##### Scanning Electron Microscopy

The sample was washed at least six times with distilled water (dist. H_2_O), suspended in 70% ethanol, and loaded on a sterile glass slide. The specimen was dried at room temperature, coated with platinum for 80 s using a platinum coater (JEC-3000FC, Joel, Tokyo, Japan), and examined using a scanning electron microscope (JSM-IT500HR, Joel, Japan) at 15 kV [24].

##### Molecular Identification

DNA was extracted according to the protocol published by Singh et al. [41]. In brief, microalgae pellets were collected by centrifugation at 4700 rpm for 10 min and washed three times. Then the pellets were lysed using 400 µL lysis buffer (4 M Urea; 0.2 M Tris-HCl, 20 mM NaCl, and 0.2 M EDTA) and 50 µL Proteinase K and incubated at 55 °C for 1 h. After incubation, prewarmed extraction DNA buffer (3% CTAB; 1.4 M NaCl; 20 mM EDTA; 0.1 M Tris-HCl; 1% Sarkosyl and mercaptoethanol) was added, and the mixture was kept in a water bath (55 °C) for 1 h. After incubation time, the mixture was allowed to cool in RT, and chloroform to isoamyl alcohol (24:1 *v*/*v*) was added. The mixture was gently mixed until a white emulsion appeared. Then, the mixture was centrifuged at 13,000 rpm for 5 min and 500 µL of upper aqueous phase was collected into a sterile Eppendorf and a double volume of 100% ethanol and 0.1 volume of 3 M sodium acetate was added. Then, the mixture was mixed by inversion and kept for 1 h at −20 °C. After 1 h, the mixture was centrifuged at 13,000 rpm for 3 min, and pellets were washed with 70% ethanol. After evaporating, the DNA was kept in 50 µL of free nuclease sterile water. The concentration of the purified DNA was evaluated using a nanodrop instrument (Genova Nano, Jenway, UK). An aliquot (2 µL) of extracted DNA was subjected to gel electrophoresis on a 0.8% agarose gel (ReadyAgarose™ Precast Gel System Bio-Rad Laboratories, Inc., Hercules, CA, USA) to check the integrity of the DNA. For 16S rRNA gene sequencing, the DNA was amplified using polymerase chain reaction (PCR) and species-specific primers (forward primer: 5′-AGAGTTTGATCMTGGCTCAG-3′; reverse primer: 3′-TACGGYACCTTGTTACGACTT-5′). Next, 7–10 µL PCR product was subjected to gel electrophoresis to confirm successful amplification. The PCR product was kept in nuclease-free H_2_O and sequenced using an ABI 3730 DNA sequencer (Thermo Fisher Scientific).

#### 2.2.3. Preparation of Microalgae Extract

Microalgae biomass was collected by centrifugation at 4700 rpm for 10 min after cultivation for 15 days. The biomass was then washed at least four times using dist. H_2_O. The wet biomass was freeze-dried by lyophilizer (LYOTRAP, LTE Scientific, Greenfield, UK) for 2 days. The dried biomass was mixed with sterilized glass balls and vortexed for 5 min to produce fine algal powder, of which 500 mg was dissolved in 500 mL dist. H_2_O and boiled at 60 °C for 30 min in a water bath (Thermo Fisher scientific). The sample was allowed to cool at room temperature (RT) and was then filtrated using Whatman filter paper No.1. The filtrate was centrifuged at 4700 rpm for 10 min to remove any algal debris and was stored at 4 °C for further application [22,23].

#### 2.2.4. Gas Chromatography–Mass Spectrometry (GC-MS) Analysis

The algal extract was prepared by mixing 163 mg *C. aeroterrestrica* BA_Chlo4 with 100 mL boiled dist. H_2_O (80 °C) and sonicating for 30 min. The specimen was allowed to macerate for 24 h before filtration using a syringe filter (0.22 µm). The filtrate was collected and dried under a vacuum at 50 °C for 48 h. After 48 h, white residues weighing 60 mg were produced. The chemical composition of the algal extract was determined using a Trace GC-TSQ mass spectrometer (Thermo Scientific, Austin, TX, USA) with a direct capillary column TG–5MS (30 m × 0.25 mm × 0.25 µm film thickness). The temperature of the column oven was 50 °C at the start and was raised by 5 °C/min to 250 °C, held for 2 min, then increased to 300 °C by 30 °C/min and held for a further 2 min. The injector and MS temperatures were held at 270 and 260 °C, respectively. Helium was utilized as a carrier gas at a constant flow rate of 1 mL/min. The solvent delay was 4 min and diluted samples of 1 µL were injected automatically using an Autosampler AS1300 coupled with GC in the split mode. Electron ionization mass spectra were collected at 70 eV ionization voltages over the range of *m*/*z* 50–650 in full scan mode. The ion source temperature was set at 200 °C. Components of the algal extract were identified by comparison of their mass spectra with those of WILEY 09 and NIST 14 mass spectral databases [42].

#### 2.2.5. Synthesis of Ag-NPs Using Algal Extract

Ag-NPs were synthesized by mixing 90 mL of 10^−3^ M silver nitrate (AgNO_3_) with 10 mL aqueous algal extract. The mixture was incubated under fluorescent light for 24 h at RT. The mixture at the beginning of the experiment was colorless then converted to pale yellow after 4 h, then to a golden-yellow color after 24 h. After 24 h, Ag-NPs were collected by centrifugation at 13,000 rpm for 30 min and washed at least three times with dist. H_2_O. Some of the washed samples were freeze-dried by lyophilizer for 6 to 8 h for biological applications, Fourier-transform infrared (FTIR) spectroscopy, energy diffraction X-ray (EDX), and mapping; others were washed three times with 70% ethanol for SEM and TEM examination; and some samples were suspended in dist. H_2_O for dynamic light scattering (DLS), and zeta potential [22,23].

#### 2.2.6. Characterization of Ag-NPs Synthesized by *C. aeroterrestrica* BA_Chlo4 (C@Ag-NPs)

##### UV-Spectrophotometry

After 24 h of biofabrication of C@Ag-NPs, an aliquot (3 mL) was examined by UV-spectrophotometer (at wavelength range 200–800 nm and a resolution of 1 nm) to detect the wavelength of the NPs.

##### FTIR Spectroscopy

The functional groups that coated the surface of C@Ag-NPs and found in the algal extract were estimated using FTIR spectroscopy (Shimadzu, Kyoto, Japan) at a spectra range of 400 to 4000 cm^−1^.

##### X-ray Diffraction Analysis (XRD)

The crystalline structure of C@Ag-NPs were detected using a D8 Advance X-ray diffractometer (Bruker, Germany). Dried powder of C@Ag-NPs was coated on an XRD grid to be estimated over 0° to 80° (2θ) using Cu K α radiation generated at 30 kV and 30 mA with scan speed of 4 deg/min.

##### EDX and Mapping Analyses

Dried powder of C@Ag-NPs was placed on clean clink paper and loaded on a carbon paste strip attached to a copper stub. Excess powder was removed by smoothly knocking the stubs. The sample was then coated using a platinum auto fine coater for 80 s at 1.8 pa and 10 mA. Finally, the EDX and mapping of the coated sample was examined by JSM-IT500HR EDX detector (STD-PC80, Joel, Japan) using SEM operation software.

##### Scanning and Transmission Electron Microscopy (SEM and TEM)

The shape and size of C@Ag-NPs was examined by SEM and TEM. Dried powder of C@Ag-NPs was placed on clean clink paper and loaded on a carbon paste strip attached to a copper stub. Excess powder was removed by smoothly knocking the stubs. The sample was coated with platinum using an auto fine coater and examined by SEM at 15 kV. For TEM, a suspension of C@Ag-NPs was sonicated for 10 min and 10 µL C@Ag-NPs suspension was dropped on a carbon-coated copper grid (300 mesh) and allowed to dry at RT for TEM examination at 120 kV (JEM-1400Flash, Joel, Japan).

##### DLS and Zeta Potential

The hydrodynamic diameter and potential charge of the C@Ag-NPs suspensions were detected using zeta sizer equipment (Malvern, UK). Briefly, C@Ag-NPs suspensions were tenfold diluted using dist. H_2_O, sonicated for 15 min, and then transferred into U-type tubes at 25 °C for measurement using a zeta sizer.

#### 2.2.7. Anticancer Activities of C@Ag-NPs

##### Cell Culture

Four malignant cell lines, including breast cancer cells MCF-7 and MDA, colon cancer cells HCT-116, and liver cancer cells HepG2, as well as two normal cell lines including human fibroblasts (HFS) and kidney cells of African green monkey (Vero), were cultured in complete DMEM and RPMI media containing 10% fetal bovine serum (FBS) and 50 U/mL penicillin and streptomycin in a 5% CO_2_ incubator at 37 °C. At 70% confluency, the cells were passaged using trypsin-EDTA and were then counted, seeded into 96-well plates at a density of 5 × 10^4^ cells/well, and incubated in a 5% CO_2_ incubator for 24 h at 37 °C [22].

##### MTT Assay

An MTT assay was used to detect the cytotoxicity of C@Ag-NPs, algal extract, Ch@Ag-NPs, and 5-fluorouracil (5-FU) against the selected cells. 5-FU and Ch@Ag-NPs (neglecting their size effect < 100 nm) were used as positive controls to approve the C@Ag-NPs activity. First, 1 mg C@Ag-NPs, Ch@Ag-NPs, and 5-FU was weighed and dissolved in 1 mL DMEM media. C@Ag-NPs and Ch@Ag-NPs were sonicated for 15 min until all particles were suspended in media, while 5-FU was vortexed for 1 min. Next, the suspensions of NPs, 5-FU, and 1 mg/mL aqueous algal extract were filtrated using a microfilter with 0.45 µm pore size. The cultured cells were then exposed to several concentrations of filtrated C@Ag-NPs (200, 100, 50, 25, 12.5, 6.25, 3.12, 1.56, and 0.78 µg/mL), algal extract (500, 250, 125, 62.5, 31.25, 15.62, 7.81, 3.90, and 1.95 µg/mL), Ch@Ag-NPs, and 5-FU (1000, 500, 250, 125, 62.5, 31.25, 15.62, 7.81, and 3.90 µg/mL) and incubated at 37 °C for 24 h. Media in the treated plates was then discarded, and 100 µL/well fresh media was added followed by 10 µL/well MTT solution (5 mg/mL), which was mixed with the media and the plates were incubated in the dark at 37 °C for 4 h. After incubation, 100 µL DMSO was added to each well to dissolve the formazan crystals and the plates were incubated on a shaker (400 rpm) for 15 min. The absorbance of each well was detected using an ELISA plate reader (Bio-Rad, USA) at 570 nm. Cell viability (%) was estimated according to the following equation

Abs_(treated)_ − Abs_(blank)_/(Abs_(control)_ − Abs_(blank)_) × 100

The IC_50_ (half-maximal growth inhibitory concentration) was calculated using a sigmoidal curve [22].

##### Inverted Light Microscope

The morphological alterations caused by IC_25_ and IC_50_ of C@Ag-NPs against MCF-7, MDA, HCT-116, and HepG2 cells were examined by inverted light microscope.

#### 2.2.8. Antioxidant Activity

The antioxidant activity of C@Ag-NPs and aqueous algal extract was examined using a DPPH assay according to the method described by Hanna et al. [43]. Briefly, 1 mg/mL C@Ag-NPs was sonicated for 10 min and then various concentrations of NPs (1000, 500, 250, 125, 62.5, 31.25, 15.6, 7.8, 3.9, and 1.95 µg/mL) were prepared. For algal extract preparation, 50 mg algal biomass powder was dissolved in 50 mL dist. H_2_O, boiled in a water bath at 60 °C for 30 min, and then various concentrations (1000, 500, 250, 125, 62.5, 31.25, 15.6, 7.8, 3.9, and 1.95 µg/mL) were prepared. Ascorbic acid was used as a reference at the same concentrations. For the antioxidant assay, 100 µL of each concentration of C@Ag-NPs or algal extract was mixed with 100 µL DPPH suspension (0.004 g DPPH powder dissolved in 100 mL absolute ethanol and then stirred for 10 min) in a 96-well plate and incubated at RT for 30 min in the dark. The absorbance of the samples, blank (ethanol only), and control (DPPH only) was read at 517 nm using plate reader and the scavenging activity was calculated according to the following equation:

Abs_(control−blank)_ − Abs_(sample−blank)_/Abs_(control−blank)_ × 100

#### 2.2.9. Antimicrobial Activity of C@Ag-NPs

##### Microbial Culture

Five bacterial strains were obtained from the Department of Microbiology, King Saud University, Riyadh, Saudi Arabia. These strains included Gram-positive bacteria (*Staphylococcus aureus* ATCC 29213, *Streptococcus pyogenes* ATCC 12344, *Bacillus subtilis* ATCC 6633) and Gram-negative bacteria (*Escherichia coli* ATCC 25922, *Pseudomonas aeruginosa* ATCC 27853). Bacterial isolates were grown in nutrient broth for up to 18 h at 37 °C and were maintained through continuous subculturing in broth and on solid media.

##### Agar Well Diffusion Method

The agar well diffusion method was employed to assess the antimicrobial activity of C@Ag-NPs, Ch@Ag-NPs, AgNO_3_, *C. aeroterrestrica* algal extract, and ciprofloxacin. Ciprofloxacin and Ch@Ag-NPs (neglecting their size effect < 100 nm) were used as positive controls to approve the C@Ag-NPs activity. Briefly, 4 mL microbial isolate was mixed with 50 mL nutrient agar media. The mixture was poured into sterilized Petri dishes and dried at 37 °C. Four 8 mm wells were created in the agar plates using a cork borer. Next, 100 µL of 500 µg/mL C@Ag-NPs, Ch@Ag-NPs, AgNO_3_, and *C. aeroterrestrica* algal extract and 5 µg/mL ciprofloxacin were applied into the 8 mm wells in triplicate and the plates were incubated for 24 h at 37 °C. Dist. H_2_O was used as a negative control. After 24 h, the diameter of the inhibition zone (mm) of each treatment was calculated using a transparent ruler [44].

##### Minimum Inhibition and Biocidal Concentrations (MIC and MBC)

The MIC and MBC of C@Ag-NPs and *C. aeroterrestrica* algal extract were assessed using a resazurin dye method according to Elshikh et al. [45]. Briefly, 100 µL nutrient broth media was added to each well of a 96-well plate from column 2 to column 12. Next, 100 µL C@Ag-NPs or *C. aeroterrestrica* algal extract (1 mg/mL) was dispensed into wells in triplicate in column 1 and various concentrations (500, 250, 125, 62.5, 31.25, 15.62, 7.8, 3.9, 1.95, and 0.98 µg/mL) were prepared across the plate to column 10 using the serial dilution method. Subsequently, 100 µL bacterial suspension (2.5–3.6 × 10^6^ CFU/mL) was mixed into each well; column 11 represented the positive control (bacterial suspension without treatment), while column 12 was the negative control (media only to monitor sterility). The plates were incubated for 24 h at 37 °C. Resazurin dye solution was prepared by dissolving 0.015 g resazurin in 100 mL dist. H_2_O, vortexing for 10 min, and filtrating using a 0.45 µm microfilter. After 24 h, 30 µL resazurin dye solution was added to each well of the plate, and the plates were incubated at 37 °C for 4 h before measuring the absorbance of each well at 570 nm using a plate reader. After 4 h, columns with no color change (blue resazurin color remained unchanged) were defined as above the MIC value. The MBC values were estimated by plating the content of wells with concentrations higher than the MIC value on nutrient agar plates. MBC values represented the minimum biocidal concentration at which no colony growth was detected on the plates.

#### 2.2.10. Statistical Analysis

All experiments were performed in triplicates, and the data are presented as mean ± SEM. One-way analysis of variance (ANOVA) was performed to compare differences between groups using graphPrism version 9.3.1 (GraphPad Software Inc., San Diego, CA, USA); *p* < 0.05 was considered statistically significant. For characterization analysis of C@Ag-NPs, origin 8 (OriginLab Corporation, Northampton, MA, USA) and ImageJ (National Institutes of Health, Bethesda, MD, USA) were utilized.

## 3. Results and Discussion

### 3.1. Morphological Appearance of Coelastrella aeroterrestrica strain BA_Chlo4

**Figure 1:**
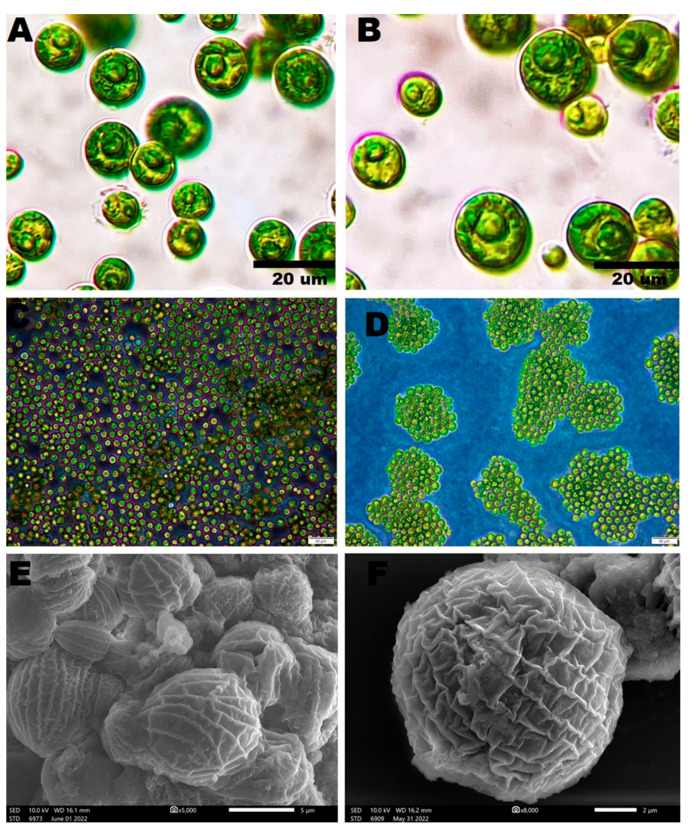
Morphological appearance of *Coelastrella aeroterrestrica* strain BA_Chlo4 under (**A**,**B**) light, (**C**,**D**) inverted light, and (**E**,**F**) scanning electron microscopes. Scale bars: 20 µm (**A**,**B**), 50 µm (**C**,**D**), and 5 and 2 µm (**E**,**F**), respectively.

The light and inverted light micrographs of novel microalgal isolate demonstrated that these algae were green, globose to broadly ellipsoidal with an average diameter of 7.6 µm, solitary, and uninucleated, with thin smooth cell walls. An obvious parietal cup-shaped chloroplast was detected in adult and young cells. Some visible granules were detected within the protoplast, while vacuoles were absent (Figure 1A–D). SEM micrographs of *C. aeroterrestrica* revealed that the algae were ellipsoidal with many irregular ribs on their surfaces (Figure 1E,F). These observations were congruent with those of Tschaikner et al., who isolated *C. aeroterrestrica* for the first time from soil in Austria [24]. Tschaikner et al. reported that the algal cells had a smooth cell wall under LM, while many meridional ribs on their surface were detected under SEM. The authors also described the structure of the chloroplast in detail, with adult microalgal cells containing a parietal, more-or-less incised hollow sphere cup-shaped chloroplast with one pyrenoid and a bi- to tripartite starch envelope, while, in young cells, the chloroplast was marginated.

### 3.2. Phylogenetic Analysis

**Figure 2:**
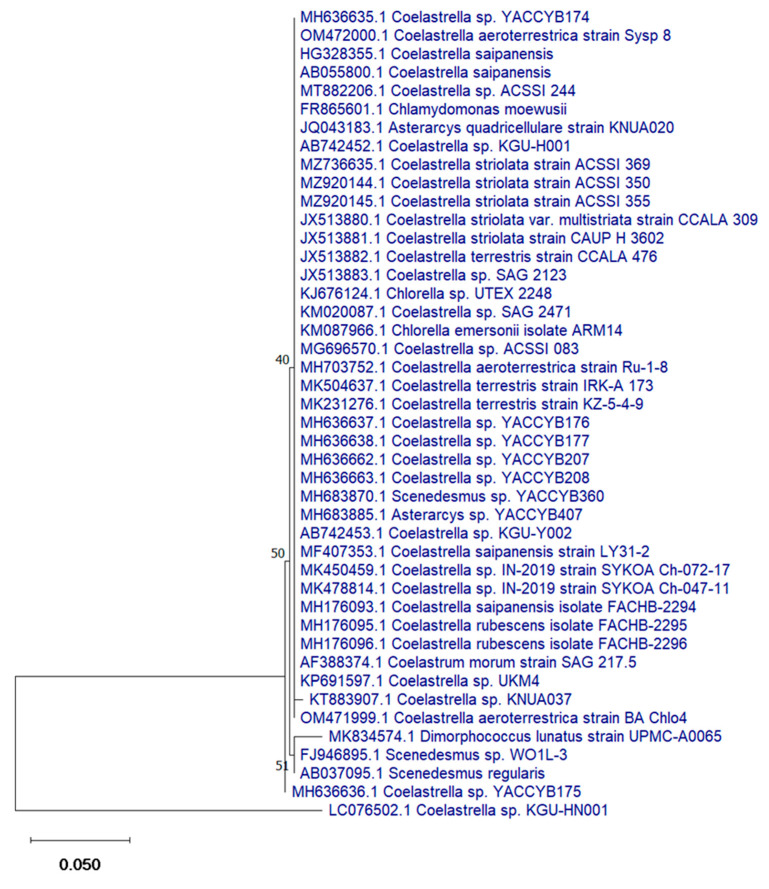
Phylogenetic tree of *Coelastrella aeroterrestrica* strain BA_Chlo4.

Phylogenetic analysis revealed that the novel isolated strain shared 100% identity with *Coelastrella aeroterrestrica strain BA_Chlo4* with 96% genomic query cover (Figure 2). The sequence of *C. aeroterrestrica* was deposited in the NCBI GenBank database under accession number ON819612.

### 3.3. GC-MS Analysis

**Figure 3:**
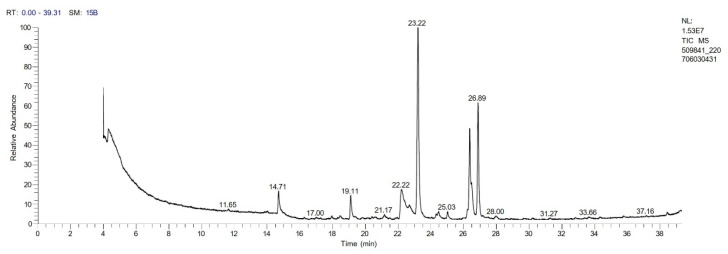
GC-MS chromatogram of *Coelastrella aeroterrestrica* strain BA_Chlo4 aqueous extract.

The chromatogram from GC-MS analysis of the algal extract exhibited 24 chromatographic peaks from 4 to 38 min, and 21 phytochemical compounds were detected (Figure 3 and Table 1). Most of these biomolecules are fatty acid (F.A ester, F.A alcohol) in nature, however, other molecules were detected including hydrocarbons, nitrogen compounds, alphatic alcohol, alkaloid, and esters. Herein, the GC-MS data demonstrated for the first time the volatile chemical components of *Coelastrella aeroterrestrica* strain BA_Chlo4. These volatile biocompounds did not represent the passivating agents, including proteins and polysaccharides, but revealed the expected capping agents for stabilizing C@Ag-NPs involving fatty acids and hydrocarbons. Ragunathan et al. performed a GC-MS analysis of methanolic extract of marine red macro algae species *Gracilaria corticata* [46]. The chromatogram showed ten distinct peaks referencing several fatty acids such as n-hexadecanoic acid, eicosanoic acid, nonanoic acid, and oleic acid; and medioresinol compounds such as bicyclo [3.2.1]oct3-en-2-one, 3,8-dihydroxy-1-methoxy-7-(7-methoxy-1,3-benzodioxol5-yl)-6-methyl-5.

### 3.4. Characterization of C@Ag-NPs

#### 3.4.1. UV-Spectroscopy

**Figure 4:**
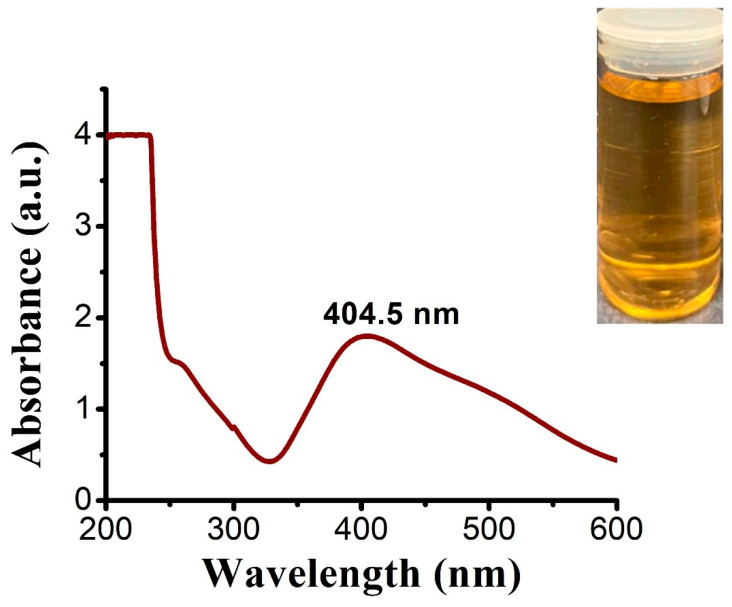
UV-spectra of Ag-NPs synthesized by *Coelastrella aeroterrestrica* strain BA_Chlo4.

UV-spectra analysis demonstrated that the maximum wavelength of C@Ag-NPs (golden-yellow suspension) synthesized by *C. aeroterrestrica* was at 404.5 nm, indicating that C@Ag-NPs have a small size and high stability (Figure 4). The optical properties of Ag-NPs are mitigated by their morphology [47]. There are different colors of Ag-NPs depending on their size and shape. Rivero et al. obtained different colors (yellow, orange, red, violet, blue, green, or brown) of Ag-NPs by changing the concentrations of reducing (dimethylaminoborane) and capping (poly (acrylic acid, sodium salt)) agents [48]. The scholars hypothesized that the change in color of Ag-NPs resulted from shape change. For instance, light yellow corresponds to a spherical shape while shifting the wavelength of 410 nm accompanied by the appearance of hexagonal, triangular, and rod shapes. Mock et al. showed that the geometrical shape of a NP plays a significant role in determining the surface plasmon resonance (SPR), while the spectrum redshifts with increasing particle size [49]. The wavelength range of 400 to 460 nm suggests the SPR of Ag-NPs [50,51]. Mora-Godínez et al. reported that the maximum wavelength of Ag-NPs synthesized using cell pellets of *Desmodesmus abundans* was at 420 nm [52]. Furthermore, Kashyap et al. screened the reduction activity of four microalgae species (*Chlorella* sp., *Lyngbya putealis*, *Oocystis* sp., and *Scenedesmus vacuolatus*) to produce Ag-NPs from their precursor (AgNO_3_). All species except *Oocystis* sp. synthesized Ag-NPs with SPR at 420 nm [50].

#### 3.4.2. FTIR Spectroscopy

**Figure 5:**
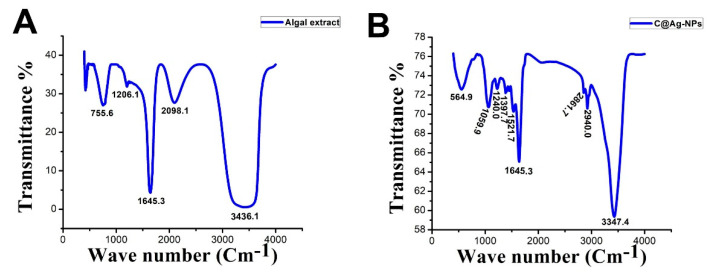
FTIR analysis of *Coelastrella aeroterrestrica* strain BA_Chlo4 algal extract (**A**) and (**B**) C@Ag-NPs synthesized by *C. aeroterrestrica*.

FTIR analysis was performed for both the algal extract and C@Ag-NPs (Figure 5). The FTIR spectra of the algal extract contained peaks at 3436.1, 2098.1, 1645.3, 1206.1, and 755.6 cm^−1^. The peak at 3436.1 cm^−1^ corresponded to a strong broad O-H stretching group of alcohol or medium N-H stretching of primary amine, while IR spectra at 2098.1 cm^−1^ referred to a strong N=C=S stretching of isothiocyanate. IR peaks at 1645.3, 1206.1, and 755.6 cm^−1^ corresponded to medium strong C=C stretching of alkene or medium N-H bending of amine, C=N stretching of imine/oxime; strong C-O stretching of vinyl or alkyl aryl ether or ester or medium C-N stretching of amine; and strong C-H bending of 1,2-disubstituted or strong C-Cl stretching of halocompound, respectively. These data indicated that the most dominant components in algal extract were proteins, while the less dominant ones were alcohol, hydrocarbon, and fatty acids. This could suggest that proteins had a main role in reducing silver nitrate into C@Ag-NPs, while other molecules such as fatty acids and hydrocarbons were responsible for stabilizing NPs. These results were consistent with the GC-MS analysis of the algal extract, in which the main components were fatty acids and hydrocarbons. The FTIR spectrum of C@Ag-NPs showed more than five peaks including 3347.4, 2940.0, 2861.7, 1645.3, 1521.7, 1397.7, 1240.0, 1059.9, and 564.9 cm^−1^. The spectra peaks were located in single bond (2500–4000 cm^−1^), triple bond (2000–2500 cm^−1^), double bond (1500–2000 cm^−1^), and fingerprint (600–1500 cm^−1^) areas. The sharp peak at 3347.4 cm^−1^ refers to a strong broad O-H stretching group of alcohol or medium N-H stretching group of primary amines. IR peaks at 2940.0 and 2861.7 cm^−1^ were related to a strong broad O-H stretching group of carboxylic acid or strong N-H stretching group of amine salts or a medium C-H stretching group of alkane. The sharpest peak at 1645.3 cm^−1^ corresponded to medium C=N stretching of imine/oxime or strong C=C stretching of alkene or medium N-H bonding of amine, while the following peak at 1521.7 cm^−1^ corresponded to strong N-O stretching of nitrocompound. The low-intensity peak at 1397.7 cm^−1^ was related to strong S=O stretching of sulfate or sulfoyl chloride or medium O-H bending of carboxylic acid or alcohol. Other peaks detected at 1240.0, 1059.9, and 564.9 cm^−1^ corresponded to strong C-O stretching of alkyl aryl ether; strong C-O stretching of alcohol or strong S=O stretching of sulfoxide; and strong C-Cl stretching of halocompound, respectively. The shift in spectra values between algal extract and C@Ag-NPs demonstrated that the surface of the C@Ag-NPs was coated with different functional groups to those detected in the algal extracts from which they emerged. Moreover, these results revealed that the main components responsible for reducing and stabilizing C@Ag-NPs using *C. aeroterrestrica* were bio-organic compounds that could be proteins or alcohols as reducing agents and fatty acids and/or hydrocarbons as capping agents.

Betül Yılmaz Öztürk extracellularly and intracellularly synthesized Ag-NPs using *Desmodesmus* sp. [53] and reported that FTIR spectra of the Ag-NPs contained peaks at 3284, 2919, 2851, 2161, 2027, 2034, 1638, 1535, 1380, 1242, 1149, 1023, 812, 717, and 551 cm^−1^. The author revealed that the FTIR peak at 2161 cm^−1^ was -S-CΞN thiocyanate, while the peaks at 2027 and 2034 cm^−1^ were -N=C=S isothiocyanate, indicating that cyanate, elemental carbon, and thiocyanate may exist within total organic carbon. Moreover, other bands related to soluble organic molecules and/or proteins were detected. Kashyap et al. screened the reduction potentiality of *Chlorella* sp. to synthesize Ag/AgCl NPs and revealed that protein and lipids had significant roles in the reduction and stabilization process of NPs [50]. In addition, Jeon et al. extracted sulfated polysaccharides (SP) from *Porphyridium cruentum* UTEX 161 and utilized it to synthesize Ag-NPs [54]. FTIR analysis of the resulting SP-Ag-NPs showed eight peaks at 3700, 3400, 2945, 1655, 1420, 1037, 881, and 724 cm^−1^, which corresponded to the stretching vibration of O−H in polysaccharide, C=O in amino acid, S=O of the sulfate group, and C−H bending, respectively. The authors reported that SP successfully reduced AgNO_3_ into Ag-NPs and capped their surface.

#### 3.4.3. XRD

**Figure 6:**
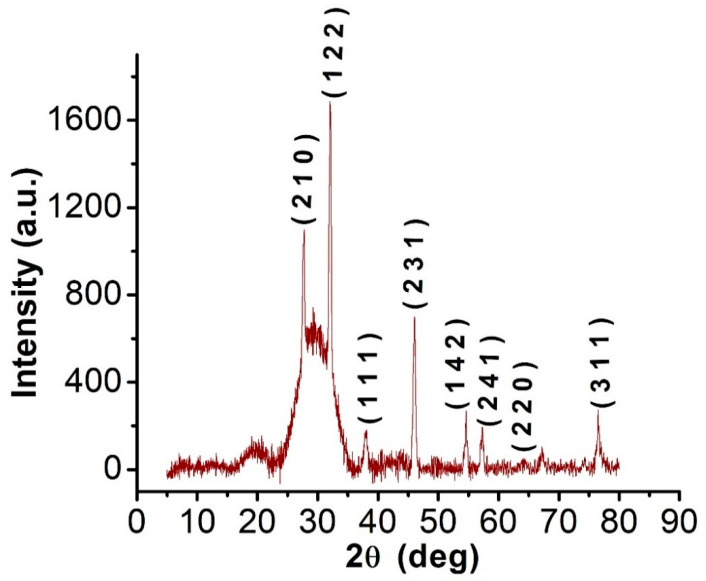
XRD graph of C@Ag-NPs synthesized by *Coelastrella aeroterrestrica* strain BA_Chlo4 strain.

The XRD graphs revealed that C@Ag-NPs exhibited 2θ values of 27.8° [55], 32.1° [56], 38.2° [57], 46.2° [58], 54.8° [57], 57.39°, 64.5° [57], and 77° [59] corresponding to (210), (122), (111), (231), (142), (241), (220), and (311) facets of silver crystals, respectively, based on the Joint Committee on Powder Diffraction Standards (Figure 6) [60]. These data indicated the crystalline nature of C@Ag-NPs and are in agreement with the findings of Ssekatawa et al., who synthesized Ag-NPs using aqueous extracts of *Prunus africana* (PAE) and *Camellia sinensis* (CSE) [57]. The reported 2θ values of PAE-Ag-NPs and CSE-Ag-NPs were 27.9°, 32.2°, 38.2°, 44.4°, 46.3°, 54.8°, 57.6°, 64.5°, and 77.4°, with 38.2°, 44.4°, 64.5°, and 77.4° attributed to silver crystal planes (111), (200), (220), and (311), respectively. XRD graph of silver NPs synthesized by *Petroselinum crispum* leaf extracts demonstrated 2θ values of 27.86°, 32.14°, 37.96°, 46.06°, 55.02°, and 57.0° corresponding to (220), (122), (111), (231), (331), and (241) planes of Ag [56].

#### 3.4.4. TEM and SEM

**Figure 7:**
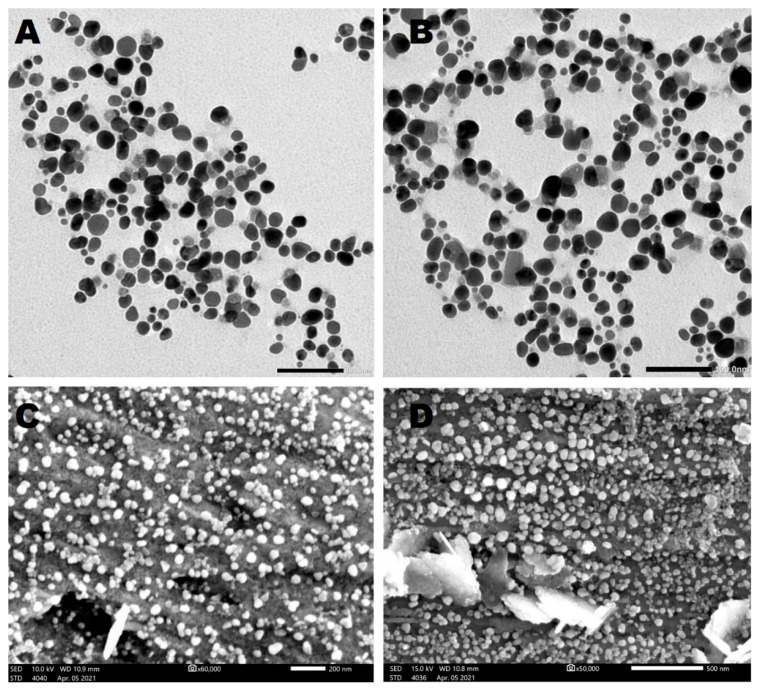
TEM (**A**,**B**) and SEM (**C**,**D**) micrographs of Ag-NPs synthesized by *Coelastrella aeroterrestrica* strain BA_Chlo4 strain. Scale bars: 100 nm (**A**,**B**), 500 and 200 nm ((**C**,**D**), respectively).

**Figure 8:**
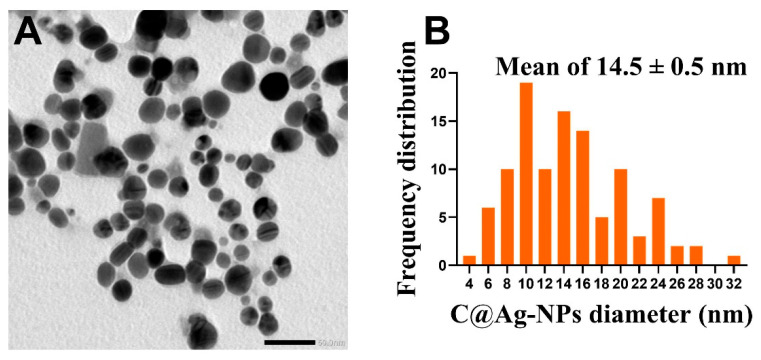
TEM micrograph of C@Ag-NPs (**A**) and frequency distribution histogram of C@Ag-NPs (**B**). Scale bar of 50 nm.

The morphology and size of Ag-NPs synthesized by *C. aeroterrestrica* were examined by TEM and SEM. TEM micrographs revealed that C@Ag-NPs have polyform shapes, including hexagonal (which was the dominant shape), quasi-spherical, rectangular, and triangular shapes (Figure 7A,B). Moreover, TEM analysis demonstrated that C@Ag-NPs were uniformly distributed without any agglomeration, implying the particles were stable. Similarly, SEM micrographs showed that C@Ag-NPs were small with an average diameter of 15.6 ± 0.6 nm and were hexagonal to quasi-spherical in shape (Figure 7C,D). The frequency distribution analysis of C@Ag-NPs revealed that C@Ag-NPs’ size range was 4 to 32 nm with an average diameter of 14.5 ± 0.5 nm, indicating the small size of C@Ag-NPs (Figure 8). These data are in correspondence with the UV-spectroscopy data, informing that C@Ag-NPs have high stability and a small size. However, the polyform distribution of C@Ag-NPs might be attributed to the formation and growth of the NPs, in which the primary seed for production of hexagonal NPs is spherical shapes [61]. Lengke et al. reported that *Plectonema boryanum* UTEX 485 successfully synthesized octahedral Ag-NPs with a size range of 5 to 200 nm [62]. Husain et al. synthesized hexagonal Ag-NPs using *Spirulina* sp. with a nanosize of 47 nm [23], while Jeon et al. showed that Ag-NPs produced by sulfated polysaccharides extracted from *Porphyridium cruentum* had a spherical shapes with an average diameter of 26 nm [54]. In addition, Husain et al. biosynthesized Ag-NPs using aqueous extract of *Nostoc muscorum* NCCU 442 and reported that they were spherical with a nanosize range of 6–45 nm and an average size of 30 nm [63].

#### 3.4.5. Mapping and EDX

**Figure 9:**
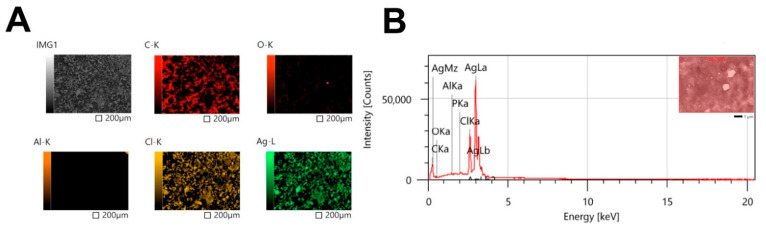
Mapping (**A**) and EDX (**B**) analysis of C@Ag-NPs synthesized by *Coelastrella aeroterrestrica* strain BA_Chlo4.

The mapping data showed that the Ag element was the dominant chemical composition in C@Ag-NPs samples (Figure 9A). The EDX analysis demonstrated a strong signal peak at 3 keV, which is a typical absorption of metallic C@Ag-NPs with a mass percentage of 80% (Figure 9B). Singh et al. synthesized Ag-NPs using *Kinneretia* THG-SQI4 extract and reported that EDX analysis of the Ag-NPs exhibited a typical optical absorption peak at 3 keV, indicating that the sample was located in the silver region [64]. In addition, other elements were detected, including Cl (9.7%), C (8%), O (1.4%), and small quantities of Al and P (Table 2). These signals could be attributed to the biomolecular corona surrounding C@Ag-NPs and derivatives from microalgae [65].

#### 3.4.6. DLS and Zeta Potential

**Figure 10:**
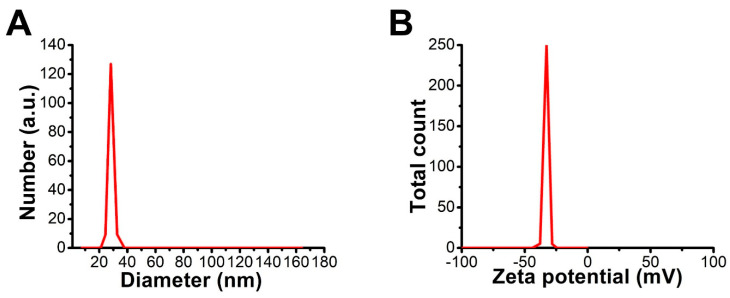
DLS (**A**) and zeta potential (**B**) graphs of C@Ag-NPs synthesized by *Coelastrella aeroterrestrica* strain BA_Chlo4.

The average hydrodynamic diameter (HD) of C@Ag-NPs was 28.5 nm, and their potential charge was ×33 mV (Figure 10A,B). The HD value of C@Ag-NPs is approximately similar to that measured using TEM micrographs, indicating the small size of C@Ag-NPs in aquatic environments with less or no agglomeration. In general, to reduce the agglomeration of NPs, high repulsion force between NPs is required. This repulsion force depends on the surface charge of the NPs. A specific value of zeta potential (>−30 mV) is recognized as desirable for an electrostatically stabilized suspension [66,67]. The high negativity value of C@Ag-NPs suggests that C@Ag-NPs are considered a colloidal stable system, and their surfaces are negatively charged. The negativity of C@Ag-NP surfaces could be attributed to the adsorption of biomolecules present in *C. aeroterrestrica* extracts onto NP surfaces, which may then play essential roles in the physical, chemical, and biological activities of C@Ag-NPs [68,69]. Farheen et al. fabricated Ag-NPs from their precursor (AgNO_3_) using aqueous extract of the *Hibiscus rosa-sinensis* plant (HRSF) and found that the HD of Ag-NPs was between 30 and 80 nm and their potential charge was equal to −25 mV, suggesting they were stabilized by HRSF biomolecules [70].

### 3.5. Toxicity of C@Ag-NPs and Algal Extract against Cancer Cells

#### 3.5.1. Antiproliferative Activity of C@Ag-NPs

**Figure 11:**
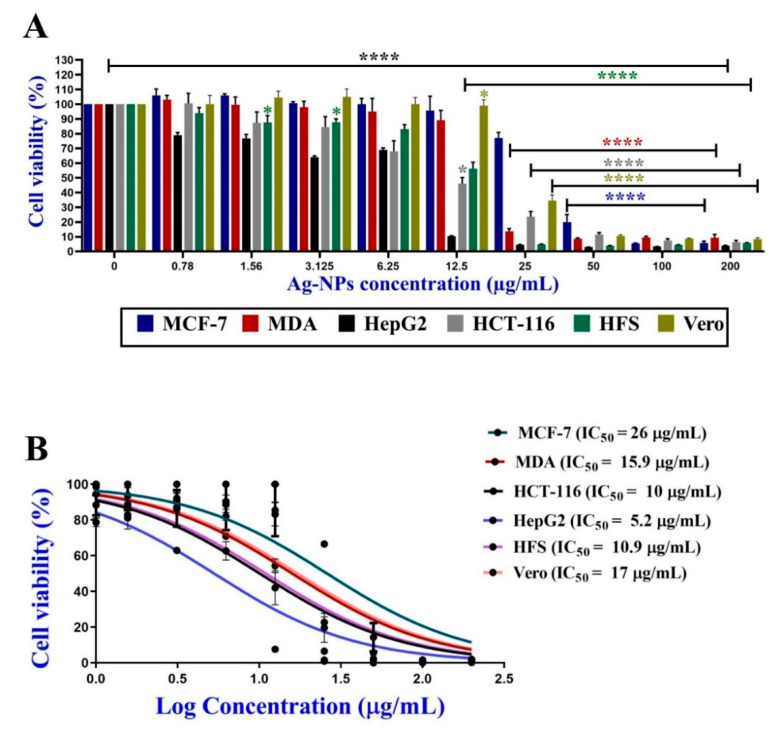
Dose-dependent growth suppression (**A**) and cell viability % (**B**) of MCF-7, MDA, HCT-116, HepG2, HFS, and Vero caused by several concentrations (200, 100, 50, 25, 12.5, 6.25, 3.12, 1.56, and 0.78 µg/mL) of C@Ag-NPs synthesized by *C. aeroterrestrica* strain BA_Chlo4. *p*-values were calculated versus untreated cells: **** *p* < 0.0001 and * *p* < 0.01.

**Figure 12:**
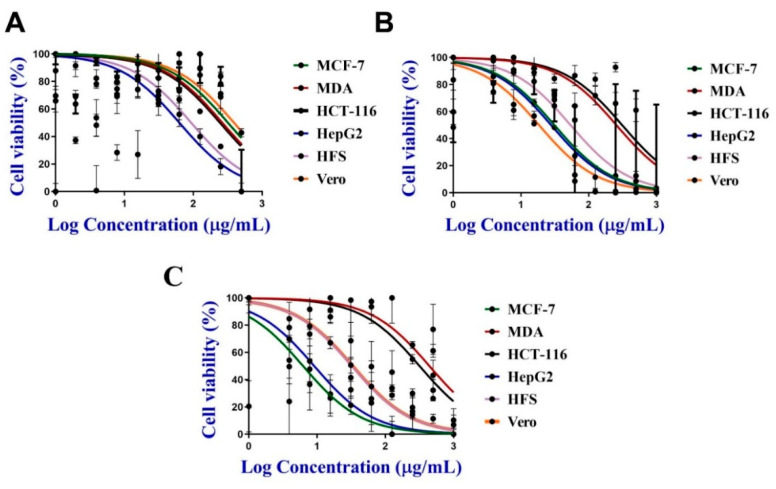
Cell viability % of MCF-7, MDA, HCT-116, HepG2, HFS, and Vero caused by several concentrations of (**A**) algal extract (500, 250, 125, 62.5, 31.25, 15.62, 7.81, 3.90, and 1.95 µg/mL), (**B**) Ch@Ag-NPs and (**C**) 5-FU anticancer drug (1000, 500, 250, 125, 62.5, 31.25, 15.62, 7.81, and 3.90 µg/mL).

The anticancer activity of 200 µg/mL C@Ag-NPs, 1 mg/mL *C. aeroterrestrica* aqueous extract, Ch@Ag-NPs, and 5-FU was screened against four malignant cell lines, including MCF-7, MDA, HCT-116, and HepG2, and two non-cancerous cell lines (HFS and Vero) using MTT assays. C@Ag-NPs significantly reduced cell viability of the four malignant cell lines in a dose-dependent manner compared with untreated cells (Figure 11A). The IC_50_ of C@Ag-NPs against MCF-7, MDA, HCT-116, HepG2, HFS, and Vero was 26.03, 15.92, 10.08, 5.29, 10.97, and 17.12 µg/mL, respectively (Figure 11B). Moreover, the IC_50_ of the algal extract against MCF-7, MDA, HCT-116, HepG2, HFS, and Vero was 319.1, 264.3, 251.5, 62.87, 93.26, and 390 µg/mL, respectively, while that of Ch@Ag-NPs was 31.18, 256.9, 312.5, 27.91, 54.06, and 18.51 µg/mL, respectively. However, the IC_50_ of 5-FU anticancer drug against the same cell lines was 56.48, 44.26, 32.14, 85.78, 32.41, and 33.11 µg/mL, respectively (Figure 12A–C and Table 3). These data indicated that C@Ag-NPs had greater antiproliferative activity against MCF-7, MDA, HCT-116, and HepG2 cells, coupled with low toxicity against non-cancerous cell lines, compared with the other tested treatments. The anticancer activity of the treatments against the tested cell lines can be expressed as C@Ag-NPs > 5-FU > Ch@Ag-NPs > algal extract. Furthermore, the most sensitive cells towards C@Ag-NPs were HepG2 followed by HCT-116. Interestingly, *C. aeroterrestrica* aqueous extract exhibited potent anticancer activity against the tested cell lines, but the activity was less than that of C@Ag-NPs against MCF-7, MDA, HCT-116, and HepG2. HepG2 cells were the most sensitive cell towards algal extract compared with the other cell lines. As expected, Ch@Ag-NPs exhibited less anticancer activity against the selected cell lines compared with C@Ag-NPs, which suggests that the smaller the size of the NPs, the greater the biological activity. Other factors may enhance the biological activities of C@AgNPs compared to Ch@Ag-NPs including high stability, less agglomeration, and the surface chemistry of C@Ag-NPs [71,72]. The most sensitive malignant cells towards Ch@Ag-NPs were HepG2 and MCF-7 cells. The variation in IC_50_ values of C@Ag-NPs, Ch@Ag-NPs, and algal extract against the tested cells could be attributed to the physiochemical features of malignant cells such as their charges that depend on metabolic state or their potentiality to interact with biomolecular corona-coated NPs or existence in algal extract or due to their resistance mechanism against drugs [73]. Hamida et al. biofabricated Ag-NPs from their precursor AgNO_3_ using *Desertifilum* sp. and assayed their anticancer activity against MCF-7, HepG2, and Caco-2 cells [22]. The IC_50_ of the Ag-NPs against MCF-7, HepG2, and Caco-2 cells was 58, 32, and 90 µg/mL, respectively. The strong toxicity of Ag-NPs was reported to be related to their charge and/or biocoat surrounding the surface of the biogenic Ag-NPs. Acharya et al. synthesized Ag-NPs (8 and 14 nm) using *Ulva lactuca* algal extract and found that the IC_50_ of the Ag-NPs against HCT-116 cells was 142 ± 0.45 µM [36].

#### 3.5.2. Morphological Appearance of MDA, MCF-7, HCT-116, and HepG2 Cells Treated with C@Ag-NPs

**Figure 13:**
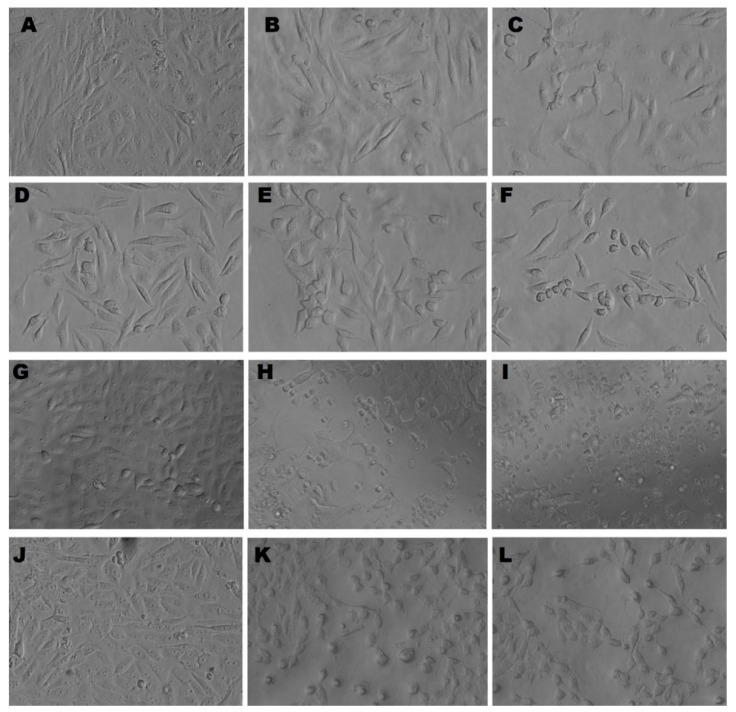
Inverted light micrographs of untreated and treated MCF-7 (**A**–**C**), MDA (**D**–**F**), HCT-116 (**G**–**I**), and HepG2 (**J**–**L**) cell lines with IC_25_ or IC_50_ of Ag-NPs synthesized by *C. aeroterrestrica* strain BA_Chlo4, respectively. Magnification was at 20×.

The inverted light micrographs showed that both the IC_50_ and the IC_25_ of C@Ag-NPs (Table 3) resulted in morphological alterations in MDA, MCF-7, HCT-116, and HepG2 cells. However, the IC_50_ of C@Ag-NPs caused the most intensive cellular changes compared with untreated cells and those treated with the IC_25_ of C@Ag-NPs. These cellular alterations included changes in cell shape, loss of cell adhesion capacity, shrinkage in cell size, reduction in a number of viable cells, and an increase in number of rounding cells (Figure 13). These results suggested that C@Ag-NPs negatively impact cellular function and morphology, inducing apoptosis in all tested cancer cells [15,22].

### 3.6. Scavenging Activity of C@Ag-NPs

**Figure 14:**
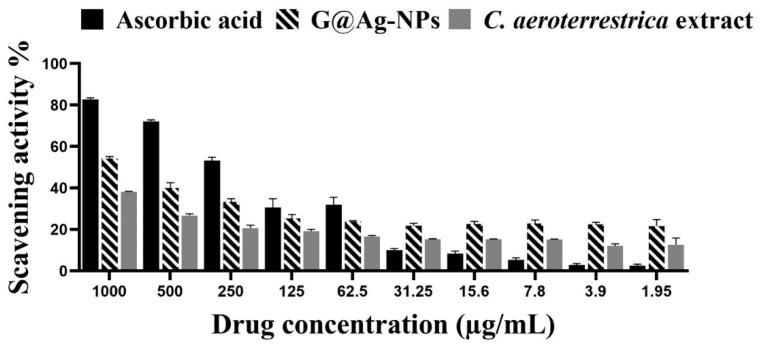
Scavenging activity (%) of C@Ag-NPs synthesized by *C. aeroterrestrica* strain BA_Chlo4 and *C. aeroterrestrica* algal extract.

The potentiality of 1 mg/mL C@Ag-NPs and algal aqueous extract to scavenge free radicals was estimated using a DPPH assay. The scavenging activity surged as the concentration of C@Ag-NPs and algal aqueous extract increased (Figure 14 and Table 4). The maximum inhibition (%) of C@Ag-NPs and algal aqueous extract was 54.2% and 38.2%, respectively at 1000 µg/mL. However, the scavenging activity of ascorbic acid (82.7 % at 1000 µg/mL) was higher compared with those of C@Ag-NPs and algal aqueous extract. These data indicated that C@Ag-NPs exhibit strong antioxidant activity compared with algal aqueous extract but show moderate antioxidant activity against free radicals compared with ascorbic acid. Husain et al. reported that Ag-NPs synthesized by *N. muscorum* and the corresponding algal extract showed a scavenging activity (maximum inhibition %) of 53.49 ± 0.73% and 12.87 ± 0.41%, respectively, compared with ascorbic acid at 56.55 ± 0.22% [63]. The authors reported that the small size and crystalline nature of Ag-NPs was a significant factor in enhancing the antioxidant activity of NPs. Hanna et al. screened the antioxidant potential of Ag-NPs produced by *Desertifilum tharense* and *Phormidium ambiguum* (D-Ag-NPs and P-Ag-NPs, respectively) and algal extracts using DPPH assays [43]. D-Ag-NPs and P-Ag-NPs exhibited a scavenging activity of 43.75% and 48.7%, respectively, while that of *D. tharense* and *P. ambiguum* was 36.14% and 33.9%, respectively. These data suggest the potentiality of D-Ag-NPs and P-Ag-NPs to scavenge the free radicals compared with the corresponding algal extracts.

### 3.7. Antimicrobial Activity

This report screened for the first time the inhibitory effect of *C. aeroterrestrica* aqueous extract and C@Ag-NPs synthesized using *C. aeroterrestrica* extract against different pathogenic bacteria. The inhibitory effect of 500 µg/mL C@Ag-NPs or algal aqueous extract was screened against *S. aureus*, *S. pyogenes, B. subtilis, E. coli*, and *P. aeruginosa* using the microdilution method. MIC and MBC values of C@Ag-NPs and algal aqueous extract against the different bacteria are reported in Table 5. C@Ag-NPs were the most potent antibacterial agent against both Gram-positive and -negative bacteria compared with algal aqueous extract. The highest MIC and MBC of C@Ag-NPs was 1.9 and 3.9 µg/mL, respectively, against *B. subtilis*, while the lowest MIC and MBC values were <0.98 µg/mL for *S. aureus*, followed by *E. coli*, *S. pyogenes*, and *P. aeruginosa* at 0.98 µg/mL and MBC of 1.9 µg/mL. These data revealed that C@Ag-NPs at the lowest concentration have marked inhibitory activity against Gram-positive and -negative bacteria. No inhibitory activity was detected for *C. aeroterrestrica* aqueous extract against all tested bacteria. Thus, the MIC and the MBC of algal extract may be above 500 µg/mL.

**Figure 15:**
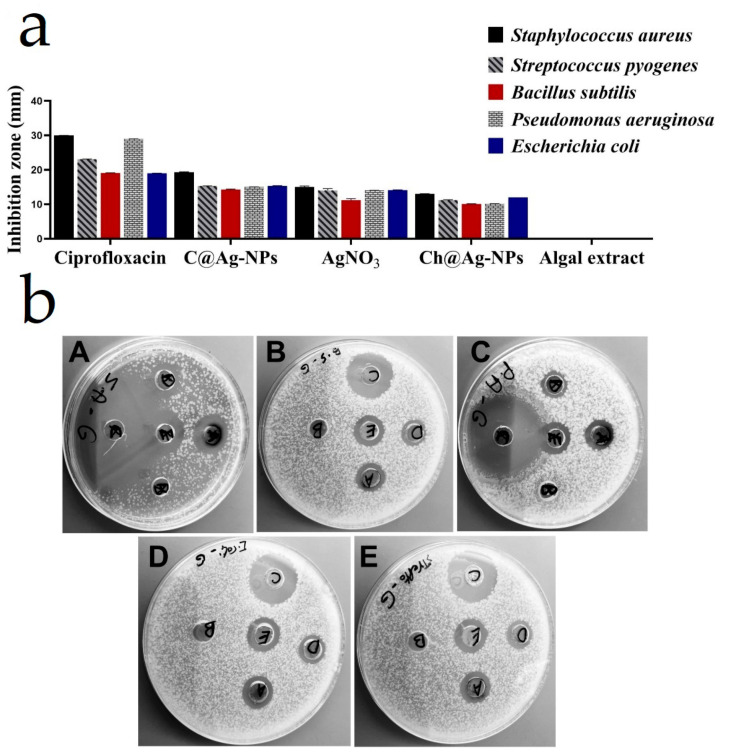
(**a**,**b**) Inhibitory effect of C@Ag-NPs synthesized by *C. aeroterrestrica* strain BA_Chlo4, algal aqueous extract, AgNO_3_, Ch@Ag-NPs, and ciprofloxacin against *Staphylococcus aureus* (**A**), *Bacillus subtilis* (**B**), *Pseudomonas aeruginosa* (**C**), *Escherichia coli* (**D**), and *Streptococcus pyogenes* (**E**). Letters on inhibition zone refer to (**A**) C@Ag-NPs, (**B**) algal extract, (**C**) ciprofloxacin, (**D**) Ch@Ag-NPs, and (**E**) AgNO_3_.

The inhibition zone diameters (IZDs) of C@Ag-NPs, algal aqueous extract, AgNO_3_, Ch@Ag-NPs, and ciprofloxacin against *S. aureus*, *S. pyogenes*, *B. subtilis*, *E. coli*, and *P. aeruginosa* are reported in Table 6. C@Ag-NPs inhibited bacterial growth to a greater extent compared with algal aqueous extract, AgNO_3_, and Ch@Ag-NPs (Figure 15). The tested concentration of algal extract (1 mg/mL) was insufficient to show a biocidal effect against the tested microbes with 0 IZD. Moreover, AgNO_3_ exhibited potent inhibitory activity against *S. aureus*, *S. pyogenes*, *B. subtilis*, *E. coli*, and *P. aeruginosa* compared with Ch@Ag-NPs. The highest IZD of 19.3 ± 0.15 mm was recorded for C@Ag-NPs against *S. aureus*. *E. coli, S. pyogenes,* and *P. aeruginosa* displayed similar responses towards C@Ag-NPs with IZDs of 15.3 ± 0.08, 15.3 ± 0.05, and 15.0 ± 0.04 mm, respectively, while the lowest IZD value induced by C@Ag-NPs was estimated against *B. subtilis* (14.27 ± 0.15 mm). For AgNO_3_ and Ch@Ag-NPs, the highest IZD was recorded against *S. aureus* at 15.0 ± 0.33 and 13 ± 0.06 mm, respectively. The lowest biocidal effect of AgNO_3_ was against *B. subtilis* with an IZD of 11.17 ± 0.44 mm, while there were even lower responses of Ch@Ag-NPs against *B. subtilis* and *P. aeruginosa* with values of 10.07 ± 0.06 and 10.1 ± 0.06 mm, respectively. The greater inhibitory activity of C@Ag-NPs against the tested bacteria compared with AgNO_3_ and Ch@Ag-NPs may be related to their small size to large surface area enabling these NPs to easily penetrate the cell membranes and interact with cellular components, subsequently resulting in bacterial death [44]. Moreover, the biomolecular corona coating the C@Ag-NPs may facilitate the conjugation between the bacterial membrane and NPs [74]. Among the tested bacteria, *S. aureus* was the most sensitive to C@Ag-NPs. This could be attributed to the potential negative charge of C@Ag-NPs enabling the NPs to interact with membranes of Gram-positive bacteria and enhancing their biocidal activity [75]. IZD, MIC, and MBC data showed that C@Ag-NPs exhibited similar negative influences on the growth of *E. coli, S. pyogenes*, and *P. aeruginosa*. This suggests that it is not only the charge of NPs that plays an important role in their antibacterial activity, but their biomolecular corona is a potential factor for enhancing the activity of NPs. Another significant factor influencing the activity of NPs is the bacterial responses and resistance mechanisms against drugs [33,35,44]. The biocidal activity of Ch@Ag-NPs was less than that of AgNO_3_ and this could be attributed to the large size of the Ch@Ag-NPs, resulting in increasing their agglomeration and trapping these NPs outside the bacterial membranes. In addition, Ch@Ag-NPs (spherical shape) showed less biocidal activity compared with C@Ag-NPs (hexagonal shape); thus, the shape of the NPs may strongly influence the release of silver ions and consequently the activity of NPs [76]. Rajamanickam et al. synthesized Ag-NPs (40–65 nm) using spirulina-associated bacterial extract and studied their antimicrobial activities [77]; the Ag-NPs had an IZD of 13 and 10 mm against *B. subtilis* and *E. coli*, respectively. Jeon et al. synthesized Ag-NPs using sulfated polysaccharides extracted from *Porphyridium cruentum* and screened their biocidal activities against *E. coli*, *B. subtilis*, *S. aureus*, and *P. aeruginosa* [54]. The SP-Ag-NPs significantly inhibited the four tested bacteria regardless of the concentration of the NPs; after incubation for 4 h, 99% of bacteria were exterminated by SP-Ag-NPs. Additionally, the inhibition rate against the tested bacteria is close to that induced by AgNO_3_ treatment.

## 4. Conclusions

The current findings report for the first time the potentiality of novel microalgae *Coelastrella aeroterrestrica* strain *BA_Chlo4* to synthesize hexagonal Ag-NPs with a small size of 14.5 ± 0.5 nm and HD of 28 nm. The resultant C@Ag-NPs showed high stability with a surface charge of −33 mV. In addition, the FTIR spectra and GC-MS analysis revealed that biomolecular corona derivatives from algal extract, including bio-organic compounds that could be proteins and hydrocarbons, or fatty acids are instrumental in reducing and stabilizing C@Ag-NPs and may enhance their biological and physicochemical properties. C@Ag-NPs exhibited significant antiproliferative activity against MCF-7, MDA, HCT-116, and HepG2 malignant cells, with low toxicity against HFS and Vero non-cancerous cells, compared with Ch@Ag-NPs, 5-FU, and algal extract. Interestingly, C@Ag-NPs displayed strong biocidal activity against all tested Gram-negative and -positive bacteria; the highest inhibitory activity was recorded against *S. aureus*. Furthermore, C@Ag-NPs have moderated potential to inhibit free radicals compared with ascorbic acid. The present findings provide a one-pot, facile synthesis method using microalgae for production of hexagonal Ag-NPs that act as potent anticancer, antibacterial, and antioxidant agents, which may have potential applications in numerous medical sectors. Further studies are needed to determine the optimum conditions for the synthesis of Ag-NPs using *Coelastrella aeroterrestrica*, aiming to increase the intensity of Ag-NPs while retaining the smaller size and stability. Moreover, the mechanistic pathway of C@Ag-NPs inside the malignant or bacterial cells should be studied to understand the pharmacokinetic nature of these NPs.

## Figures and Tables

**Table 1:** Biomolecules of *Coelastrella aeroterrestrica* strain BA_Chlo4 aqueous extract analysis by GC-MS.

No.	Biomolecule Name	Retention Time	Area %	Mentioned Factor	Molecular Formula	Molecular Weight	Structure
1	1-Heptanol	4.10	0.31	738	C_7_H_16_O	117	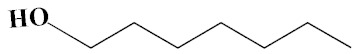
2	2-Ethyl-1-hexanethiol	4.14	0.25	648	C_8_H_18_S	146	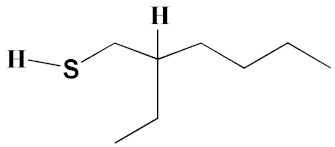
3	1-Octene	4.35	4.58	637	C_8_H_16_	112	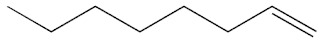
4	1-Deoxy-d-mannitol	5.05	0.46	635	C_6_H_14_O_5_	166	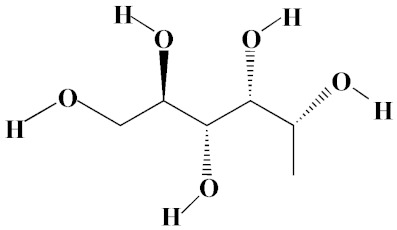
5	1-Chlorotetradecane	11.65, 14.01	0.51, 0.40	583, 614	C_14_H_29_Cl	232	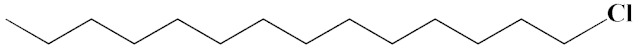
6	Methyl 10-methylundecanoate	14.71	3.76	816	C_13_H_26_O_2_	214	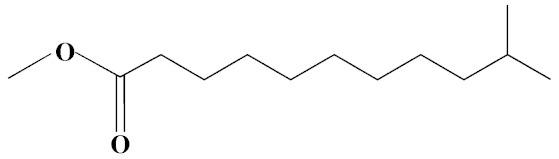
7	1-Nonadecene	17.95	0.52	704	C_19_H_38_	266	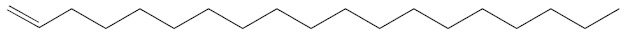
8	2-Methylhexadecan-1-ol	18.46	0.50	684	C_17_H_36_O	256	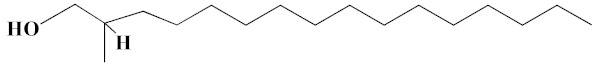
9	Arachidic acid methyl ester	19.11	3.68	906	C_21_H_42_O_2_	326	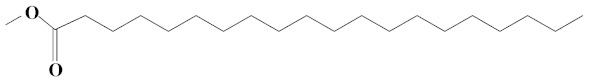
10	Oxiraneundecanoic acid, 3-pentyl-, methyl ester, *cis*-	20.42	0.33	651	C_19_H_36_O_3_	312	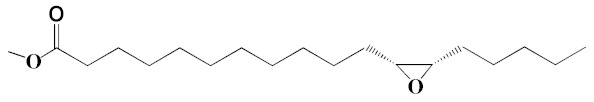
11	Methyl 12-methyltetradecanoate	21.17, 24.48	1.10, 1.43	685, 671	C_16_H_32_O_2_	256	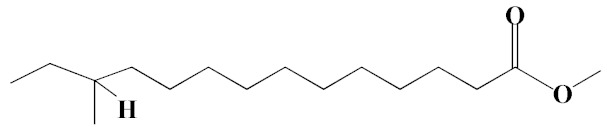
12	Caffeine (3,7-Dihydro-1,3,7-trimethyl-1H-purine-2,6-dione)	22.19, 22.69	6.12, 0.97	891, 830	C_8_H_10_N_4_O_2_	194	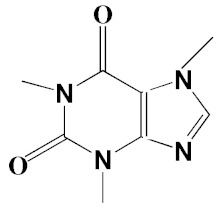
13	Methyl palmitate (Hexadecanoic acid, methyl ester)	23.23	34.13	930	C_17_H_34_O_2_	270	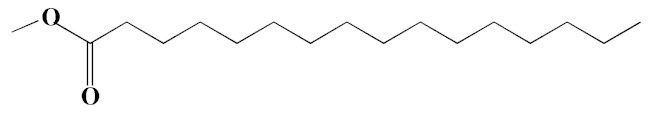
14	Methyl juniperate (Methyl 16-hydroxy-hexadecanoate)	24.33	0.64	689	C_17_H_34_O_3_	286	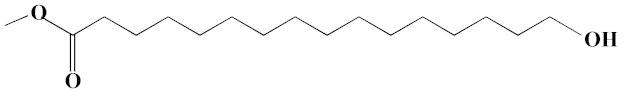
15	Cyclopentanetridecanoic acid, methyl ester	25.02	1.27	753	C_19_H_36_O_2_	296	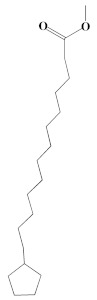
16	E,E-11,14-Octadecadienoic acid, methyl ester	26.23	0.43	779	C_19_H_34_O_2_	294	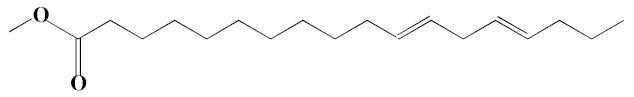
17	Oleic acid, methyl ester	26.38, 26.49	14.18, 4.61	902, 871	C_19_H_36_O_2_	296	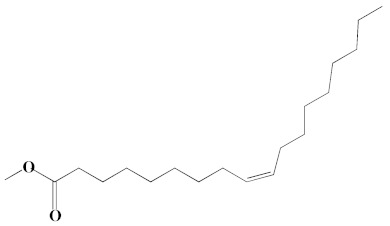
18	Methyl stearate	26.89	18.50	900	C_19_H_38_O_2_	298	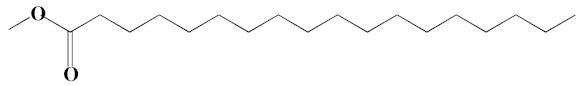
19	2E,15Z-14-Methyl-2,15-octadecadien-1-ol	27.99	0.66	658	C_19_H_36_O	280	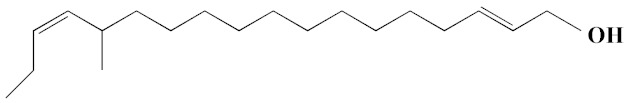
20	Dotriacontane	38.43	0.65	674	C_32_H_66_	450	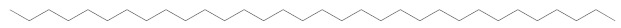

**Table 2:** Elemental compositions of C@Ag-NPs synthesized by *Coelastrella aeroterrestrica* strain BA_Chlo4.

Element	Line	Mass%	Atom%
C	K	8.01 ± 0.02	37.05 ± 0.08
O	K	1.49 ± 0.02	5.17 ± 0.07
Al	K	0.21 ± 0.01	0.44 ± 0.02
P	K	0.40 ± 0.01	0.72 ± 0.02
Cl	K	9.79 ± 0.02	15.34 ± 0.04
Ag	L	80.10 ± 0.1	41.27 ± 0.05
Total		100.00	100.00

**Table 3:** IC_25_ and IC_50_ of C@Ag-NPs, *C. aeroterrestrica* aqueous extract, Ch@Ag-NPs, and 5-FU against the selected cell lines.

Cells	Drugs (µg/mL)							
	C@Ag-NPs		*C. aeroterrestrica* Aqueous Extract		Ch@Ag-NPs		5-FU	
	IC_25_	IC_50_	IC_25_	IC_50_	IC_25_	IC_50_	IC_25_	IC_50_
MCF-7	13.015	26.03	159.55	319.10	15.59	31.18	28.24	56.48
MDA	7.96	15.92	132.15	264.3	128.45	256.9	22.13	44.26
HCT-116	5.04	10.08	125.75	251.5	156.25	312.50	16.07	32.14
HepG2	2.645	5.29	31.435	62.87	13.955	27.91	42.89	85.78
HFS	5.485	10.97	46.63	93.26	27.03	54.06	16.205	32.41
Vero	8.56	17.12	195.00	390.00	9.255	18.51	16.555	33.11

**Table 4:** Inhibitory activity (%) of C@Ag-NPs synthesized by *C. aeroterrestrica* strain BA_Chlo4 and *C. aeroterrestrica* algal extract against free radical DPPH.

Concentrations (µg/mL)	Ascorbic Acid	C@Ag-NPs	*C. aeroterrestrica* Extract
1000	82.7 ± 0.8	54.2 ± 1.0	38.2 ± 0.2
500	72.2 ± 0.7	40.0 ± 2.5	26.7 ± 0.9
250	53.3 ± 1.6	33.4 ± 1.4	20.7 ± 1.4
125	30.6 ± 4.2	25.4 ± 1.8	19.2 ± 0.9
62.5	31.9 ± 3.5	23.9 ± 0.4	16.7 ± 0.4
31.25	10.1 ± 0.7	22.8 ± 1.2	15.4 ± 0.2
15.6	8.4 ± 1.2	22.6 ± 1.3	15.4 ± 0.1
7.8	5.4 ± 0.9	22.9 ± 1.8	15.2 ± 0.2
3.9	2.9 ± 0.7	22.5 ± 0.9	12. 6 ± 0.9
1.95	2.5 ± 0.8	21.7 ± 3.1	12.7 ± 3.1

**Table 5:** Minimum inhibition and bactericidal concentrations (MIC and MBC) of C@Ag-NPs (µg/mL) and algal aqueous extract against *Staphylococcus aureus*, *Streptococcus pyogenes*, *Bacillus subtilis*, *Escherichia coli*, and *Pseudomonas aeruginosa*.

Microbes	Treatments					
	*C. aeroterrestrica* Extract (µg/mL)			C@Ag-NPs (µg/mL)		
	MIC	MBC	MIC/MBC	MIC	MBC	MIC/MBC
*Staphylococcus aureus*	>500	>500	1.0	<0.98	<0.98	1.0
*Escherichia coli*	>500	>500	1.0	0.98	1.95	0.5
*Pseudomonas aeruginosa*	>500	>500	1.0	0.98	1.95	0.5
*Streptococcus pyogenes*	>500	>500	1.0	0.98	1.95	0.5
*Bacillus subtilis*	>500	>500	1.0	1.95	3.9	0.5

**Table 6:** Inhibition zone diameter (mm) of C@Ag-NPs (µg/mL), algal aqueous extract, AgNO_3_, Ch@Ag-NPs, and ciprofloxacin against *Staphylococcus aureus*, *Streptococcus pyogenes*, *Bacillus subtilis*, *Escherichia coli*, and *Pseudomonas aeruginosa*.

Microbes	Inhibition Zone Diameter (mm)				
	C@Ag-NPs	*C. aeroterrestrica* Extract	AgNO_3_	Ch@Ag-NPs	Ciprofloxacin
*Staphylococcus aureus*	19.3 ± 0.15	0.0 ± 0.0	15.0 ± 0.33	13.0 ± 0.06	30.0 ± 0.03
*Escherichia coli*	15.3 ± 0.08	0.0 ± 0.0	14.1 ± 0.11	12.0 ± 0.0	19.03 ± 0.03
*Pseudomonas aeruginosa*	15.0 ± 0.04	0.0 ± 0.0	14.0 ± 0.04	10.1 ± 0.06	29.0 ± 0.03
*Streptococcus pyogenes*	15.3 ± 0.05	0.0 ± 0.0	14.03 ± 0.51	11.2 ± 0.11	23.1 ± 0.05
*Bacillus subtilis*	14.27 ± 0.15	0.0 ± 0.0	11.17 ± 0.44	10.07 ± 0.06	19.07 ± 0.06

## Data Availability

The data supporting this article are shown in Figure 1, Figure 2, Figure 3, Figure 4, Figure 5, Figure 6, Figure 7, Figure 8, Figure 9, Figure 10, Figure 11, Figure 12, Figure 13, Figure 14 and Figure 15 and sex-tables. The datasets analyzed in the present study are available from the corresponding author upon reasonable request.
